# WHO working group meeting to develop WHO Recommendations to assure the quality, safety and efficacy of enterovirus 71 vaccines

**DOI:** 10.1016/j.vaccine.2020.05.001

**Published:** 2020-07-06

**Authors:** Dianliang Lei, Elwyn Griffiths, Javier Martin

**Affiliations:** a World Health Organization, Geneva, Switzerland; b Kingston upon Thames, United Kingdom; c National Institute for Biological Standards and Control, United Kingdom

**Keywords:** Enterovirus vaccines, Development, Manufacture, Quality control, WHO recommendations

## Abstract

Enterovirus A71 (EV71) is one of the major causative agents of hand, foot and mouth disease (HFMD), and is sometimes associated with severe central nervous system syndromes. Vaccines against EV71 infection have been developed or are in development in several countries and few have been licensed in China. In response to requests from some of these countries, WHO convened a working group meeting in Shanghai China from 11 to 12 September 2019 to develop WHO Recommendations to assure the quality, safety and efficacy of EV71 vaccines. Meeting participants included members of the drafting group, experts from vaccine developers, manufacturers, regulators and academia. The epidemiology of EV71, as well as the development, regulation and standardization of EV71 vaccines were reviewed in the meeting. Information on R&D, manufacturing, quality control and standardization of EV71 vaccines was presented by vaccine developers, manufacturers and regulators. Based on their experience, the working group discussed the main principles that would determine WHO’s position on quality, safety and efficacy of EV71 vaccines. The working group agreed to develop WHO Recommendations to assure the quality, safety and efficacy of inactivated EV71 vaccines with a scope covering only whole virus inactivated vaccines. Other type of vaccines, such as EV71 virus-like particles (VLPs) will not be covered as they are still at the developmental stage. The outline of the document was agreed and will follow the usual style of WHO recommendations. It was also agreed to submit the draft Recommendations for review and adoption to the WHO ECBS in 2020 following discussion at a WHO informal consultation, which will include NRAs and vaccine manufacturers.

## 1 Introduction

Enterovirus A71 (EV71) was first isolated and characterized from cases of neurological disease in California in 1969. It is the major cause of hand, foot and mouth disease (HFMD) and is sometimes associated with severe central nervous system (CNS) diseases [1], [2], [3]. Outbreaks of EV71 have occurred throughout the world including some serious epidemics particularly in the Asia-Pacific region. EV71 infection causes a range of effects, from asymptomatic infection, to mild HFMD, severe complications with CNS, and cardiopulmonary failure. Several vaccines against EV71 virus are under development and three inactivated EV71 vaccines have already been licensed in China. Some National Regulatory Authorities (NRAs) requested the development of WHO recommendations to guide NRAs and vaccine manufacturers in the development, manufacture and evaluation of the quality, safety and efficacy of EV71 vaccines. In response to this request, WHO is planning to develop such recommendations for EV71 vaccines which will provide guidance to vaccines manufacturers and NRAs. Based on this International Standard document, WHO could prequalify this vaccine and enable UN agencies and other organizations to purchase the vaccine to prevent EV71 infection.

A working group including experts from the NRAs of EV71 vaccine producing countries, academia, other experienced NRAs and representatives from industry was convened from 11 to 12 September 2019 in Shanghai, China to review the development and regulation of these vaccines and discuss issues related to their quality, safety and efficacy.

The meeting was opened by Dr Dianliang Lei (WHO) who outlined its objectives which were to review the development and standardization of EV71 vaccines, discuss a proposed outline of future WHO Recommendations on EV71 vaccines and agree the next steps and timelines**.** Mr Xiangyu Wang, Director the Division of International Organizations, Department of International Cooperation of National Medicinal Products Administration welcomed participants drawing attention to the importance of standardization for the quality, safety and efficacy of vaccines and to the fact that China now had a new vaccine law encouraging Chinese manufacturers to export vaccines according to international standards. Dr Miao Xu, Deputy Director, Institute of Biological Products Control of National Institutes of Food and Drug Control (NIFDC), also welcomed all experts to the meeting on behalf of the NIFDC, which was hosting the meeting in collaboration with the WHO.

Following consideration of the declaration of interests of participants according to WHO procedure and the announcement that its legal department had judged these not to be an impediment to all those present from participating in the meeting, Dr Lei proceeded to announce that the meeting would be chaired by Dr P Minor (WHO Consultant) and that Drs E Griffiths (WHO Consultant) and J Martin (National Institute of Biological Standards and Control (NIBSC)) would act as rapporteurs. Dr Minor then welcomed participants who were all invited to introduce themselves.

The meeting remit was to discuss issues relevant to the quality, safety and efficacy of EV71 vaccines and agree on the scope, outline and content of future WHO recommendations for EV71 vaccines. All issues were discussed in the open session of the meeting with all participants present, although the final decision on the way forward towards developing the WHO Recommendations, its structure, scope and time table were discussed in a closed session of the drafting group alone.

## 2 Background

Dr Lei briefly described the background to the current working group meeting which had been organized to develop WHO Recommendations for EV71 vaccines. He explained that the WHO had played a key role for 70 years in establishing International Biological Reference Materials necessary to standardize biological materials as well as developing WHO guidelines and recommendations to assure the quality, safety, and efficacy of biological products. These norms and standards were based on scientific consensus achieved through international consultations and designed to assist WHO Member States ensure the quality and safety of biological medicinal products and related in vitro biological diagnostic tests worldwide. This work was accomplished through its biologicals programme coordinated by a Secretariat at WHO, Geneva, the WHO Expert Committee on Biological Standardization (ECBS) and WHO Collaborating Centres for Biological Standardization. Reports of the ECBS are published annually in the WHO Technical Report Series (e.g. 2017, TRS 1011; 2018, TRS 1016).

Dr Lei also introduced EV71 and the problems of HFMD worldwide. HMFD is a common infectious disease caused by a group of enteroviruses, including Coxsackie A16 (CA16) and EV71 viruses. EV71 infection causes a range of effects, from asymptomatic infection, to mild HFMD, to infection with severe complications involving the CNS and cardiopulmonary failure and with mortality rates as high as 82–94% in severe cases. Although outbreaks of EV71 occur throughout the world, including some serious epidemics in Asia, Europe and America, since 2008 the numbers of HFMD cases have increased significantly in China to the extent that it is now considered a major public health issue. A Guide to Clinical Management and Public Health Response for Hand, Foot and Mouth Disease (HFMD) was produced by WHO Regional Office for the Western Pacific (WPRO) in 2011 to guide experts and clinicians in the management of the infections and diseases [4].

EV71 infection is known to be vaccine preventable. Several EV71 vaccines are at different stages of development with three already licensed in China. The development of combined EV71 and CA16 vaccines is also on-going with candidates at the pre-clinical stage of development. It was pointed out that International Reference Standards to aid the development and quality control of EV71 vaccines had already been established by the ECBS: the 1st International Standard for Anti EV71 Serum Human (1000 IU/ampoule) plus a low titre 1st International Reference Reagent for EV71 neutralization assays with an assigned value of 300 IU/ampoule. Chinese National Standards for EV71 vaccines had also been established. In addition, the 1st International Standard for EV71 inactivated vaccine was to be proposed for establishment to the ECBS in October 2019. However, no written international standards exist although there had been requests from NRAs and vaccine manufacturers for WHO recommendations to assure the quality, safety and efficacy of EV71 vaccines and the ECBS had proposed their development in 2016 [5]. It was considered that EV71 vaccines are needed to mitigate or prevent outbreaks of HFMD and this might involve WHO prequalification.

Further background to EV71 infection was provided by Dr P Minor who noted that such infections were rarely severe in Europe and North America but that in China this virus was causing extremely severe disease with high mortality in young children. Although HFMD is caused by a number of different enteroviruses, in the past CA16 has caused most cases but EV71 has caused the most severe cases. HFMD is mainly a disease of young children but sero-prevalence of EV71 antibodies can be much higher in adults than infants implying silent circulation of the virus and the current focus on vaccine development. EV71 is a member of the Picornovirus family, a positive single stranded RNA virus, with three different genotypes (A, B C) and at least 11 sub-genogroups (A, B1-5, C1-5). C4 is the commonest in China with C1 and C2 genogroups predominating in Europe and B4 and B5 in other regions.

## 3 EV71 vaccine development, standardization and regulatory evaluation

Dr M Xu (NIFDC) presented an overview of progress in research and development of EV71 vaccines, highlighting key points and again noting that HFMD is the most serious public health problem for children in China in recent years. It was reported that the development of inactivated whole EV71 virus vaccine and vaccines based on virus-like particles (VLP) was being carried out in different countries around the world. In China, three whole-virus inactivated vaccines had already been approved, marketed and were in use. Several multivalent vaccines both inactivated and VLP, were also under development, a bivalent vaccine (EV71/CA16), a trivalent vaccine (EV71/CA16/CA6) and a tetravalent vaccine (EV71/CA16/CA6/CA10). In all these cases the vaccine viruses were grown in Vero cells or human diploid cells, and recombinant vaccine antigens were expressed in Sf9 cells (an insect cell line) or yeast.

Dr Xu reported that the screening and evaluation of virus strains used for vaccine production had been carefully carried out leading to the selection of production strains with the potential to produce vaccines which would induce cross protecting antibodies. Two animal models had been developed to study EV71 vaccines. One is the suckling monkey (rhesus) model, which enables studies on EV71 infection and pathogenicity, and the suckling mouse model, which can be used to evaluate vaccine immunogenicity. The protective efficacy of EV71 vaccines had been shown using both animal models. It was also reported that a number of reference materials for HFMD vaccines were available. A National Standard of EV71 Antigen based on the C4 sub-genogroup, as well as a National Standard of EV71 neutralizing antibody was available in China, as was a National reference for potency of EV71 vaccines. In addition, and as previously noted, the 1st International Standard for anti-EV71 serum (human) had been established by the ECBS in 2015 and work was ongoing to develop the 1st International Standard for EV71 inactivated vaccine (*this was subsequently established by the ECBS at its meeting in October 2019*). National Standards of CA16 antigen and for CA16 neutralizing antibody were also available in China and National Standards for CA6 were under development.

The safety and efficacy of inactivated EV71 vaccines had been confirmed in large phase III clinical trials. Dr Xu reported that vaccine efficacy was at least 90% against EV71-associated HFMD and 100% against EV71- associated HFMD with neurologic complications [6], [7], [8], [9], [10]. The consistency of immunogenicity among all vaccine lots had also been investigated and found appropriate. In addition, the results of cross-reaction studies with 5 prominent epidemic sub-genogroups had shown broad cross-neutralizing activity was induced by the C4 EV71 vaccines. Cross-reaction of neutralizing antibody responses had been confirmed in EV71 vaccinated children using a pseudovirus-based neutralization assay with 12 sub-genogroups pseudovirus which cover the 5 epidemic sub-genogroups.

Large-scale vaccine production was now in place in China and Dr Xu described one such production process. This involved growth of EV71 virus either in Vero or human diploid cells in a bio-reactor followed by micro and ultrafiltration, virus concentration, purification using column chromatography and inactivation. The inactivated product was then further purified prior to formulation and filling. This process resulted in a product with over 95% purity and, for Vero cell derived vaccine, with residual DNA level of < 10 pg/dose.

Dr Xu concluded that such advanced and stable vaccine production systems guaranteed the quality and stability of vaccine production and over 65 million doses of vaccine had been released since 2016. According to a rough estimate, inactivated EV71 vaccine had been safely used in 25 million children in China since licensure, resulting in a dramatic reduction of severe EV71 infection and deaths. It was reported that the cases of HFMD deaths in China in 2018 decreased by 93.08% compared with the average value in 2010–2015.

A number of vaccine developers and manufacturers then described the development and production of their own EV71 vaccines, some of which had completed Phase III studies and were already licensed, whereas others were at an earlier stage of development. Dr X Li (China National Biotechnology Group Co Ltd, Shanghai) reported on the development and production of their inactivated EV71 vaccine which had been licensed in China. Considerable effort had been placed on screening and finding a EV71 C4 production strain with strong immunogenic potential and genetic stability. Production was based on growth of the EV71 virus in Vero cells. Following harvesting, the virus was purified using various ultrafiltration steps and chromatography prior to inactivation using formaldehyde. There was further purification following inactivation and prior to formulation with aluminium hydroxide adjuvant. Two animal models, a neonatal and mature mouse model, were used for characterizing product potency and immunogenicity and antigen content was estimated using an ELISA. Clinical studies, including a pivotal Phase III multicentre randomized double-blind placebo controlled study, showed the inactivated vaccine to be highly protective against EV71 associated HFMD, especially against severe disease, and with no safety issues [8]. The results of cross reaction studies showed the vaccine induced broad cross neutralizing antibodies. There was no cross-protection against CA16 or other enteroviruses associated with HFMD. This vaccine is now licensed in China and post-market studies are on-going. Long lasting immunity (up to 5 years) following the priming vaccination has been reported with no significant adverse events to date.

Dr Y Hu (Sinovac) reported on the development and production of Sinovac’s inactivated EV71 vaccine which had also been licensed in China. Considerable attention had again been given to strain selection and the strain chosen for vaccine production was a high yielding C4 genogroup showing uniformity of the VP1 sequence during passage. It was able to produce vaccine generating high titre antibody, showing good protection in animal challenge studies, as well as wide ranging cross protection. The production process flowchart described showed EV71 virus inoculation and propagation in Vero cells, followed by harvesting and inactivation by formaldehyde. This was followed by purification involving a series of Dia-filtration, precipitation, sucrose gradient centrifugation and sterile filtration steps prior to formulation with aluminium hydroxide adjuvant. Pre-clinical studies of Sinovac EV71 vaccine included safety evaluation (acute toxicity test, local stimulation test, allergy test, repeat toxicity study (in both rat and Cynomolgus monkey) as well as immunogenicity studies in mice (immune dosage, ED_50_) and rats (immunization schedule, longevity of serologic responses). Protection had been evaluated using a neonatal mouse model developed by the University of Sydney which involved immunization of the mother and challenge of the new born mice. Passive protection was also investigated. The clinical evaluation from Phase I to III was described with Phase III studies based on a multi-centre, randomized, double-blinded, placebo-controlled studies with an immunization schedule of Day 0 and 28 (400U/dose). Altogether, the study involved 10,000 subjects. Confirmed cases of EV71 infection were based on a preliminary diagnosis involving clinical symptoms plus PCR results followed by DSMB review of symptoms, duration, PCR results, virus isolation and neutralizing antibody testing. Protection rate (%, 95% CI) against EV71-associated HFMD was found to be 94.6% and against EV71-associated hospitalized and severe HFMD 100% [9]. Work is currently on-going to define the immunological surrogate of protection. There were no significant differences in safety between the vaccine and placebo group, the major adverse effect being transient fever mainly after the first dose. Despite natural infection consistently promoting neutralizing antibody increases in the placebo group, the seropositive rate and GMT were reported to be significantly higher in the vaccine group over a five-year period. The evaluation of the immunogenicity and safety of the simultaneous administration of EV71 vaccine (dose 1) with recombinant hepatitis B vaccine (Hep B) on day 1 and EV71 vaccine (dose 2) with group A meningococcal polysaccharide vaccine (Men A) on day 30 was found not to be inferior to separate administration of each vaccine. The Sinovac EV71 vaccine production capacity had now been built up to more than 20 million doses/year. In view of its cross reactivity with different genogroups of EV71 it is believed this EV71-C4a vaccine could be used worldwide.

The research and development of another already licensed inactivated EV71 vaccine was described by Professor Q Li (Institute of Medical Biology). This vaccine is produced using human diploid cells KMB17. Following virus seed isolation, selection and adaptation to growth in human diploid cells, master and working virus seed banks of a C4 sub-genogroup were established. Analyses of biological characteristics, immunogenicity, genetic characteristics and stability were conducted prior to the final selection of the strain for use in production. An ELISA method for detecting the antigen content was established, and the safe dose of the antigen determined through dose–effect analyses in immunized animals, which were also used for the evaluation of vaccine efficacy. Vaccine production involved virus culture on KMB17 diploid cells and virus harvesting followed immediately by inactivation. Inactivated virus was then subjected to ultrafiltration, purification and further ultrafiltration prior to the addition of aluminium hydroxide adjuvant and formulation. The virus inactivation process was validated through the establishment of inactivation kinetic curves and a production process using formaldehyde for 72 h established. Sensitive cells are used for testing for effective inactivation and indicator viruses used to evaluate the cell sensitivity of each inactivation procedure. Quality control of the final product includes an identity test (an ELISA to show the product contains EV71 virus antigen) and a potency test (antigenic content, ED_50_ in an animal model and neutralizing antibody titre determination). Specifications had been set for these various parameters. A Macaque animal model of viral pathogenesis had been established and all pathological parameters used to evaluate immune protective effects of the experimental vaccine were obtained using this model. Phase III clinical studies showed the diploid cell derived EV71 vaccine to be safe and effective in clinical use. The results showed that the neutralizing antibodies induced by the vaccine were able to neutralize most of sub-genogroups. High neutralizing antibody titres were found in most cases except for a weak neutralization of the C1 genotype [10]. No strong inflammatory reactions were induced. It was reported that the safety and clinical protective efficacy of the vaccine had also been assessed in a phase IV clinical study using passive surveillance and follow up of HFMD cases. During 13 months of case monitoring the accumulated cases collected from the control group were 29 compared with 1 in the vaccine group. A total of 947 adverse events were recorded during 13 months of the study of which 35 were considered to be vaccine related. Most of the adverse effects were identified as HFMD caused by other enteroviruses.

Dr J Liu (Beijing Minhai Biotechnology Co) reported on the development of a recombinant EV71 vaccine produced in the yeast *Hansenula polymorpha*. The wild type strain was obtained from ATCC and modified by gene knock-out to an uracil deficient strain. A recombinant expression vector was constructed to express the P1 protein of the C4a sub-genogroup of EV71 virus and transferred into the host cell by electroporation. Following selection and culturing, a high expression strain was obtained for use in production. Biological and growth characteristics were defined including the sequencing of the EV71 P1 gene incorporated into the yeast chromosome. Tests included foreign gene sequencing, copy number, genetic stability and antigen yield. Production is based on the usual master and working seed system. Process development consisted of fermentation, cell disruption, purification and formulation to include an aluminium adjuvant. The antigen was fully characterized by peptide mapping, molecular weight, amino acid composition, N/C terminal sequencing and disulphide bond content, including analysis of post-translational modifications (glycosylation, phosphorylation, acetylation). Protein characteristics were reported to have been evaluated using a range of techniques including transmission electron microscopy, dynamic light scattering, isoelectric point, analytical ultracentrifugation, UV spectrophotometry, differential scanning calorimetry and fluorometry. High level structure was examined using circular dichroism and cryo-electron microscopy. Specifications were developed from these various parameters. Nonclinical immunogenicity studies were carried out in mice and showed good neutralizing antibody responses lasting at least 26 weeks after two doses of 2 µg of recombinant EV71 vaccine. The neutralizing antibody titre after the second dose was significantly higher than after the first dose. Cellular immunity had also been explored and showed that the levels of IFN-γ, IL-2, IL-5 and IL-6 stimulated by recombinant EV71 vaccine were significantly higher than the levels in the adjuvant controls. Challenge protection studies in one day old suckling mice showed that good protection was afforded by passive immunity as well as by maternal antibody. Toxicity studies carried out in mice, rats, guinea pigs and monkeys highlighted no particular safety signals and a clinical trial certificate was granted in 2018.

Dr J Lee and Dr E Jung described the research and development in the Republic of Korea of a bivalent inactivated whole virus vaccine against both EV71 and CA16 viruses with the aim of blocking infection by these pathogens. As in the development of the other vaccines described above, considerable attention was paid to vaccine strain selection and characterization. Both EV71 and CA16 were grown in Vero cells and screened for adventitious viruses using the usual range of in vivo tests (adult mice, suckling mice, embryonated eggs, guinea pigs) and for human, porcine, bovine, simian and other respiratory viruses using qPCR. The CA16 virus was also screened using a 28-day in vitro adventitious agent assay (Vero, MRC-5, MDCK). The manufacturing process was based on growth in a bioreactor using a carrier system (200L for EV71 and 1000L for CA16) and serum free media. Following virus harvesting, subsequent downstream processing involved affinity and Ion-exchange chromatography. It was mentioned that size exclusion chromatography (SEC) and ultra-purification processes were excluded to enable easier process scale-up in the future. Inactivation of the purified viruses involved the use of a chemical reagent other than formaldehyde. This, it was claimed, better maintained the natural immunogenicity of the viruses. Quality control included virus identification using RT-PCR, VP1 antigen concentration, general protein concentration, residual infectivity test, potency (in vivo) as well as testing for endotoxin and host cell impurities.

Preclinical evaluation included monovalent EV71 and CA16 dose finding studies which were carried out in mice and rabbits involving MNT and virus specific IgG, as well as bivalent vaccine studies looking for possible immune interference and adjuvant (aluminium hydroxide) effects. Bivalent efficacy studies were carried out in neonate mice using passive transfer. Virus challenge studies were also carried out in gerbils. In addition, a range of single and repeat toxicology studies were reported. The vaccine is currently being evaluated in a blinded, randomized placebo controlled Phase I study in healthy humans in Korea.

The small scale early development of an inactivated EV71 vaccine in Thailand was described by Dr S Phumiamorn (Thailand). She indicated that since EV71 is known as a pathogen which may cause severe complications and death in infected children, it had been considered that developing vaccine against EV71 virus in Thailand and for use in Thailand would be important. It had also been decided that an inactivated product would be the best approach to take. The purpose of the study was therefore to provide a foundation for the development of inactivated EV-71 whole virus vaccine from a sub genogroup C4 EV-A71 strain in Thailand by developing preliminary small-scale virus production and purification processes. The first step was the preparation of an EV71 virus stock.

The sub-genogroup C4 EV71 (THA-08–29961) strain had been isolated from a fatal case of severe HFMD in Thailand in 2008. This strain was used to prepare the virus stock by propagation in confluent monolayers of Rhabdo myosarcoma cells (RD cells). After virus propagation in RD cells with many sub-passages, a single plaque was picked and inoculated onto another monolayer of cells. EV71 virus-infected cell cultures were identified by indirect fluorescence using monoclonal antibody to EV71.

Production of EV71 prototype vaccine was on a Vero cell monolayer (ATCC CCL-81) in roller bottles. A three-day culture in roller bottles provided sufficient virus for further study. Following harvest, the EV71 virus was inactivated using 0.01 or 0.02% formaldehyde for up to 10 days at either 4 °C or 37 °C. Results showed that no infectious virus could be detected even after 24hr at 37 °C but that live virus could be detected after 10 days when inactivation was conducted at 4 °C using 0.02% formaldehyde. Following inactivation, the product was purified using tangential flow filtration and a series of sucrose density ultracentrifugation steps. The resulting product was tested for Vero cell protein and residual DNA and shown to be of high purity. In addition, immunogenicity studies in BALB/C mice had shown that this product induces neutralizing antibodies against Thai EV71. The response was considerably improved with the inactivated EV71 product with alum. This prototype product will now be developed further as an EV71 vaccine candidate.

Dr H Yang (CDE) reported on the regulatory evaluation of EV71 vaccines in China. To put this into perspective, she summarized the vaccine licensing processes in the country by describing the current organizational structures for drug and vaccine administration and supervision of vaccine review regulations. She also discussed the recent reforms of the review and approval processes for new drugs and vaccines that had taken place in China. This had involved among other things increasing the number of reviewers of the National Medical Products Administration (NMPA) in order to improve capacity and to speed up the review process. The CDE now has a staff of over 700 employees and 19 offices, amongst them the Office of Pharmaceutical Science of Biological Products, Office of Clinical Evaluation of Biological Products, as well as an Office of Biostatistics and Clinical Pharmacology. The CDE has, among other duties, the responsibility for the acceptance and technical review of applications for drug clinical trials and drug marketing authorization, and for the technical review of drugs involved in emerging medical products, such as regenerative medicine. In addition, it is responsible for coordinating the inspection, testing and other work related to drug review. The Office also participates in the drafting of laws, regulations and normative documents related to the registration and administration of drugs, including the formulation and implementation of drug review norms and technical guidelines. Many guidelines are available relating to the regulatory system for vaccines.

Because the traditional registration pathway could take over 6 years from submission to decision-making regarding licensing or not, major reforms had been introduced since 2015 to make the system faster, to reduce backlog and to better align the registration process with ICH and other international guidelines, following good review practices. Particular attention had been paid to the review and decisions-making processes related to clinical efficacy. Accelerated and priority review pathways had been introduced to improve the time of evaluation of new drugs and to encourage innovation. A special review and approval system has also been introduced to deal with products for use in for example public heath emergencies. In addition, provisions had been put in place to improve communications during critical steps of innovative drug development with the aim of jointly solving difficult problems and issues not covered by technical guidelines. Some of these new processes were used in the evaluation of the candidate EV71 vaccines and their supporting clinical trial data.

Dr Q Mao from NIFDC and Dr J Martin from NIBSC gave presentations on the quality control and development of reference materials. Dr Mao focused on efforts conducted by NIFDC on the development of norms and standards to support the development and quality control of EV71 inactivated vaccines in China. NIFDC’s work had been essential during the pre-marketing phase of currently licensed products and is also helping to assess these vaccines in the post-marketing phase. An overview of EV71 vaccines in development was given which included not only the licensed inactivated vaccines in China but also current attempts to develop VLP vaccines and even a live-attenuated vaccine being developed in Japan. Vaccines against enterovirus serotype CA16, also associated with large HFMD outbreaks, were briefly mentioned, as were EV71/CA16 bivalent vaccines in early development. Chinese national standards for EV71 neutralizing antibody and EV71 antigen had been established by NIFDC during the pre-marketing phase. They were established following collaborative studies conducted by NIFDC and the three main vaccine manufacturers in China and contributed to ensuring the accuracy, comparability and repeatability of anti-EV71 neutralizing antibody and EV71 antigen detection assays and hence to EV71 vaccine standardization. NIFDC, in collaboration with the NIBSC, also contributed to the establishment of WHO International Standards for anti-EV71 serum and EV71 antigen. In addition, NIFDC developed in vivo assays in mice to measure the immunogenicity of EV71 vaccines which allows the comparison of in vivo potency between different vaccine products. In vivo efficacy studies using an immunization-challenge model in suckling mice were also conducted. Correlation between immunogenicity and efficacy in vivo was observed for the three EV71 vaccine products currently licensed. Although in vivo assays remain to be further standardized, these studies conducted by NIFDC greatly helped vaccine development and design of clinical trials in humans. Further efforts are underway to unify and standardize the quality control of EV71 vaccines in China using selected methods and references and introducing new assays such as nucleotide sequencing of vaccine seeds and analysis of virus particle purity by HPLC. NIFDC is also actively supporting the post-marketing quality control of EV71 vaccines. Technical key points for the quality control and evaluation of EV71 vaccines were published; a new national reference for vaccine potency was developed and detailed trend analyses to determine production consistency are regularly conducted.

Dr J Martin summarized key aspects related to EV71 vaccine standardization including details of recently developed WHO International Standards, the main points to be considered for the preparation of the WHO Recommendations and the possible use of surveillance activities for monitoring the impact of EV71 vaccination on enterovirus circulation in humans. New methods for the sensitive direct detection and typing of all enterovirus serotypes from clinical and environmental samples were presented. These have allowed the detection of the silent circulation of multiple enterovirus serotypes in different parts of the world, including new genotypes of EV71 and CA16 in Pakistan where no major HFMD outbreaks have been described. It was proposed that such methods be used to analyze sewage samples in areas of EV71 vaccine use to analyze its impact on enterovirus ecology, particularly on the molecular epidemiology of EV71 and other serotypes causing HFMD. A proposed structure for the new WHO Recommendations for EV71 vaccines was discussed, largely based on the WHO Recommendations for inactivated poliomyelitis vaccines (IPV) but acknowledging the differences between the two types of products. Key aspects to include in the WHO document were reviewed such as the need to establish a seed lot system, the importance of virus purification procedures and the choice of EV71 strain/genogroup for neutralization assays used to assess clinical studies. Establishing, monitoring and documenting effective and consistent virus inactivation procedures is an essential requirement. However, some manufacturers appear to deviate from the standard control procedures used for inactivated whole virus vaccines such as IPV (see below). In addition, Dr Martin gave full details of the establishment of WHO International Standards for anti-EV71 serum and EV71 inactivated vaccine antigen completed through collaborative studies coordinated by NIBSC and NIFDC. A concern was identified related to the nature of the viral particles in EV71 vaccines with potential major impact on the standardization of in vitro and in vivo potency assays. In contrast to IPV, which only contains full virus particles, inactivated EV71 vaccines contain full virus particles as well as a significant proportion of empty particles. There is evidence that full and empty virus particles differ in their immunogenic and antigenic properties which means that estimating vaccine production consistency will require understanding these differences and being able to measure the full to empty virus particle ratio of different vaccine products and lots.

## 4 General discussion and proposed outline of WHO Recommendations for EV71 vaccines

Discussion of the various presentations raised several points for clarification and identified key points for the Recommendations document. These were:

1) Scope: inactivated EV71 vaccine or EV71 vaccine plus CAV 16, 6,10 etc, VLPs
2) Production issues such as inactivation before or after purification, filtration, tests for effective inactivation
3) Full/empty inactivated viral particles; how to measure the ratio, and does it matter
4) Assays: ED_50_, antigen assays, animal models, reagents, monoclonal antibodies
5) Cross protection: do you need to show it and if so how?
6) Clinical end-points of clinical trials: HFMD and severe disease
7) Surveillance (Phase IV studies).

It was agreed that the WHO Recommendations for EV71 vaccines should be based on the model of the WHO Recommendations for inactivated polio vaccines (IPV) with an introduction relating to EV71 vaccines and then following the sections, style and topics of the IPV document. The Introduction should cover epidemiology, disease burden, causative agents, genotypes/genogroups and types of vaccines. It was also agreed that the scope of the Recommendations would cover only inactivated EV71 vaccines since only such vaccines had been licensed to date. However, parts of the recommendations might also serve as guidance for the inactivated Coxsackie virus vaccines in development and the section on clinical evaluation as a guide for EV71 vaccines based on virus like particles.

The issue of whether inactivation should take place prior to or after virus purification was considered to be important and would need careful management in the text since vaccines produced by both approaches are already licensed. The concern here is safety and relates to the potential for active virus to remain within aggregates or particles in newly harvested cultures and not inactivated by the formaldehyde (or other agent). This is the lesson learnt from the Cutter incident with inactivated polio vaccine. In the WHO Recommendations for IPV and Hepatitis A vaccines, there are recommendations to filter the virus suspension prior to and after inactivation to remove particles where there is a potential for active virus to remain. The possibility of over inactivation of the purified virus was also raised and should be considered and controlled. The test used to monitor effective inactivation (detection of residual infectivity) also needs attention as manufacturers appear not to be measuring the same human dose equivalent volume as for IPV (1,500 human doses for IPV). Likewise, the issue of full or empty viral particles should be discussed, as would the best way of measuring this ratio. There was also a need to discuss whether this mattered clinically.

The various assays, such as ELISAs, antigen assays, animal models, reagents and monoclonal antibodies would need to be expanded in the Recommendations as well as guidance on what QC tests should be carried out by National Control Laboratories. The clinical evaluation section of the Recommendations (Part C) would need to discuss the clinical trial endpoint, whether this should be HFMD or more severe disease. Methods of surveillance in phase IV studies would also need airing as would the need to show cross-protection. Cross-protection had been studied in animals in the licensed inactivated vaccines in China and in immunized human sera [11], [12].

## Disclaimer

The author is staff member of the World Health Organization. The authors alone are responsible for the views expressed in this article and they do not necessarily represent the decisions, policy or views of the World Health Organization.

## Declaration of Competing Interest

The authors declare that there is no conflict of interest regarding the publication of this article.
